# Biomedical Applications of Functionalized Composites Based on Metal–Organic Frameworks in Bone Diseases

**DOI:** 10.3390/pharmaceutics17060757

**Published:** 2025-06-08

**Authors:** Chenxi Yun, Zhe Yuan, Rim El Haddaoui-Drissi, Ruitong Ni, Yunyun Xiao, Zhenhui Qi, Jie Shang, Xiao Lin

**Affiliations:** 1 Key Lab for Space Biosciences and Biotechnology, School of Life Sciences, Northwestern Polytechnical University, Xi’an 710072, China; chenxiyun@mail.nwpu.edu.cn (C.Y.); yzhe@mail.nwpu.edu.cn (Z.Y.); rimdrissi@mail.nwpu.edu.cn (R.E.H.-D.); qi@nwpu.edu.cn (Z.Q.); 2 Queen Mary University of London Engineering School, Northwestern Polytechnical University, Xi’an 710072, China; jp2023304329@qmul.ac.uk; 3 Institute of Chemistry and Biochemistry, Freie Universität Berlin, 14195 Berlin, Germany; xiaoy93@zedat.fu-berlin.de; 4 Shenzhen Research Institute, Northwestern Polytechnical University, Shenzhen City 518063, China

**Keywords:** metal–organic frameworks, bone diseases, bone regeneration, biomedical applications

## Abstract

Every year, millions of people worldwide suffer from bone tissue damage caused by bone trauma and surgical operations, as well as diseases such as osteoporosis, osteoarthritis, osteomyelitis, and periodontitis. Bone defect repair is one of the major challenges in the field of regenerative medicine. Although bone grafts are the gold standard for treating bone defects, factors such as donor sources and immune responses limit their application. Functionalized nanomaterials have become an effective means of treating bone diseases due to their good biocompatibility and osteoinductivity, anti-inflammatory, and antibacterial properties. Metal–organic frameworks (MOFs) are porous coordination polymers composed of metal ions and organic ligands, featuring unique physical properties, including a high surface area–volume ratio and porosity. In regenerative medicine, MOFs function as the functions of drug carriers, metal ion donors, nanozymes, and photosensitizers. When combined with other functional materials, they regulate cellular reactive oxygen species, macrophage phenotypic transformation, bone resorption, osteogenesis, and mineralization, providing a new paradigm for bone tissue engineering. This study reviews the classification of functionalized MOF composites in biomedicine and the application of their synthesis techniques in bone diseases. The unique in vivo and in vitro applications of MOFs in bone diseases, including osteoarthritis, osteoporosis, bone tumors, osteomyelitis, and periodontitis, are explored. Their properties include excellent drug loading and sustained release abilities, high antibacterial activity, and bone induction abilities. This review enables readers to better understand the cutting-edge progress of MOFs in bone regeneration applications, which is crucial for the design of and functional research on MOF-related nanomaterials.

## 1. Introduction

Bone is a highly dynamic balanced structure that involves the destruction of old bone and the regeneration of new bone [1]. It is composed of various functional cells, including osteoblasts, osteoclasts, endothelial cells, minerals, and extracellular matrix, endowing bones with weight-bearing, movement, and endocrine balance functions [2]. The problem of bone defect regeneration occurs not only in the context of bone trauma and surgical operations but also in bone diseases, including osteoporosis, osteoarthritis (OA), osteomyelitis, and periodontitis [3]. Importantly, these diseases are frequently associated with defects in the femur and alveolar bones, which present unique clinical challenges due to their structural and functional significance. Osteoporosis, for instance, is a systemic metabolic disorder characterized by reduced bone mass and bone microarchitecture deterioration, which significantly weaken bones, such as the femur and tibia, thereby increasing the risk of fractures and impairing load-bearing function [4]. OA is a degenerative joint disease characterized by cartilage degradation, subchondral bone remodeling, and synovitis, primarily affecting joint areas such as the knee and hip [5]. Osteomyelitis is a bacterial infection that commonly affects bones, such as the femur and tibia, leading to localized inflammation, bone necrosis, and chronic infection that complicates healing processes [6]. Bone tumors—including primary malignancies, such as osteosarcoma, and secondary metastatic lesions originating from cancers, such as breast and prostate cancer—predominantly form destructive osteolytic or osteoblastic lesions in bones, such as the femur, humerus, and tibia, compromising structural integrity and leading to severe functional deficits [7]. In addition, periodontitis is a chronic inflammatory condition triggered by bacterial biofilms that affects the alveolar bone surrounding teeth in the maxillofacial region, resulting in progressive tissue destruction and eventual tooth loss if untreated [8]. Due to the limited regenerative capacity of autologous bone, bone repair needs to be carried out through means such as autologous and allogeneic bone transplantation and tissue engineering. Autologous grafts have the disadvantages of difficulty acquisition and limited quantity [9]. Therefore, the development of functionalized nanomaterials and their modified allogeneic grafts has become an effective means for treating bone diseases. Functionalized bone materials need to have good biocompatibility, osteoinductivity, and antibacterial, anti-inflammatory, and immunomodulatory functions [10].

Metal–organic frameworks (MOFs) are porous coordination polymers with periodic network structures composed of metal ions and polydentate organic ligands [11,12]. These organic ligands also serve as linkers and coordinate with metal ions to form one-, two-, or three-dimensional networks. Generally, the ligands used in MOF organic linkers can be classified as nitrogen-centered ligands (pyridyl, imidazolyl, cyano, etc.), oxygen-centered ligands (aliphatic or aromatic carbolic acid, phosphonate, sulfonate, etc.), and other functional group-based linkers [13,14,15]. Therefore, the porosity and structure of a MOF can be tuned by decorating the ligand structures or using various metal ions. The distinctive structure properties of MOFs, including a high area–volume ratio and porosity, have attracted widespread attention. MOFs have not only been widely applied in gas storage, catalysis, sensors, and membranes [16,17,18,19,20] but also have revolutionary applications in the biomedical field, including as drug carriers, biological nanozymes, and photosensitizers, and in the treatment of cancer, bone disease, kidney disease, and so forth [21,22,23,24,25].

Recently, MOFs have been increasingly harnessed to address complex challenges in the treatment of bone disease. For instance, divalent MOFs, such as ZIF-8, HKUST-1, and Mg-MOF-74 have good stability and degradation kinetics, making them excellent drug carriers and controlled metal ion donors [26]. Catalytic MOFs, such as MIL-100 and Mn-TCPP, can simulate enzyme activity to drive Fenton-like reactions and generate reactive oxygen species (ROS) for therapeutic purposes, while photosensitive MOFs can integrate ligands, like tetrakis-(4-carboxyphenyl) porphyrin (TCPP), to achieve photodynamic therapy (PDT) for antibacterial and antitumor applications [27]. These biomedical functions, ranging from drug delivery to ROS modulation, position MOFs as versatile platforms for bone regeneration and disease management. By bridging the gaps in mechanical, biological, and therapeutic aspects, MOFs can provide innovative solutions for bone tissue engineering.

This review introduces the application classifications of MOFs in the biomedical field, including drug carriers, metal ion donors, nanozymes, and phototherapeutic agents. We also review the application of functionalized nanomaterials based on MOFs in bone regeneration for bone defects, OA, osteoporosis, bone tumors, osteomyelitis, and periodontitis. The biological regulatory effects of different types of MOFs in these diseases in the forms of nanoparticles, hydrogels, membranes, surface coating, and 3D printing, are discussed. This provides a therapeutic paradigm for the design of bone functional materials based on MOFs.

## 2. Classification of MOF Applications in Biomedicine

**Figure 1:**
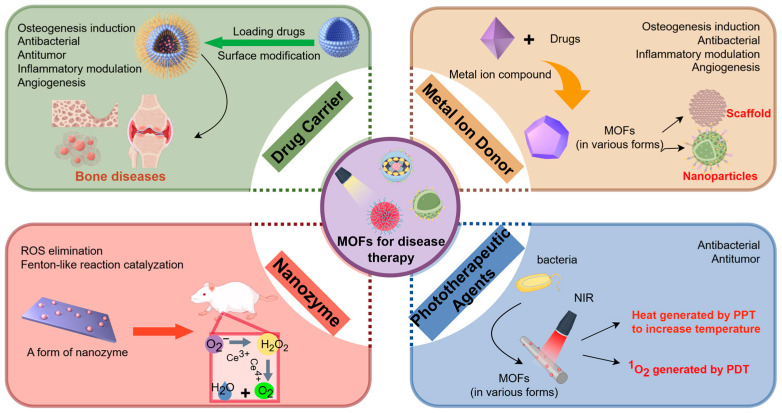
The classification of MOFs applications in biomedicine includes drug carriers, metal ion donors, nanozymes, and phototherapeutic agents. The picture is made by Figdraw.

Due to the diversity of MOFs in terms of their chemical properties, size, preparation, and modification, with the diversification of synthesis and modification methods, MOF-based composites are increasingly being applied in the field of life sciences. Many excellent reviews summarizing and analyzing the application of MOFs in biology have been published, some of which are important in research advances. In this section, MOFs are classified into four types—drug carriers, metal ion donors, nanozymes, and phototherapeutic agents (Figure 1)—according to their primary roles in disease therapy and the impact of their functionalization on osteogenesis, antibacterial activity, and biocompatibility.

### 2.1. Drug Carriers

Since metal carboxylate-based MOFs were reported as pioneering examples of loading and releasing ibuprofen in drug delivery systems, MOFs have been recognized as excellent drug carriers due to their high porosity, large surface area, high load capacity, and customizable structure [12,28]. Using MOFs as drug carriers has attracted considerable interest in biological application. For instance, MOFs have been used to encapsulate and release therapeutic agents (e.g., BMP-6, dexamethasone, metformin, and quercetin) to treat bone regeneration (promoting osteogenic differentiation via PI3K/AKT pathways), skull defect repair, OA (reducing inflammation and cartilage degradation), and osteosarcoma (inducing immunogenic cell death and inhibiting tumor growth) [29,30,31,32,33]. The advantages of MOFs in drug delivery include improving therapeutic efficacy by stabilizing drugs with short half-lives and targeted delivery by stimulating reactivity or tissue-specific design [12]. Drugs can be loaded into MOFs via adsorption, encapsulation, non-covalent interactions, and covalent bonding [34]. These strategies can be achieved during MOF synthesis either through one-pot encapsulation (incorporating the drug directly into the framework) or post-synthetic modification (loading the drug into a preformed MOF) [35]. The drug release process mainly depends on the degradation of MOFs in response to environmental stimuli, such as pH [36], light [37,38], and temperature [39]. However, as exogenous nanoparticles (NPs), MOFs still face challenges, such as immunogenicity and macrophage phagocytosis, which reduce drug delivery efficiency and trigger inflammation. To address these issues, surface modifications using biocompatible molecules, such as polyethylene glycol (PEG) or stem cell membranes (SCMs), as well as optimizing particle size, shape, and surface charge, have proven to be effective strategies [40]. However, the partial metal ions and organic ligands used to construct MOFs can be toxic to health and the environment or show weak solubility. Therefore, the properties of MOFs, including their toxicity, water solubility, lipophilicity, structure homogeneity, and so on, should be considered to meet the medical requirements.

### 2.2. Metal Ion Donors

Metal ions play vital roles organisms, controlling many biological functions; signal transmission, osteogenic activity, enzyme catalysis, and the release of metal ions through biomaterials can activate related functions [41,42,43]. MOFs constructed through coordination between metal ions and organic ligands can control the release of metal ions during stimulus-responsive degradation [44]. When MOFs are prepared using biologically active metal ions, and the sustained release of metal ions (e.g., Zn^2+^, Mg^2+^, Cu^2+^) can provide therapeutic benefits for antibacterial, anti-inflammatory, and bone tissue engineering or exert systemic therapy in combination with other drugs [45,46,47,48]. For example, Zn^2+^ (from Cu-Mg-MOF, ZIF-8 composites, SCM/ZIF-8, etc.) promotes osteogenesis, angiogenesis, antibacterial activity, and anti-inflammatory effects [49,50], while Mg^2+^ (Cu-Mg-MOF, PLGA/Exo-Mg-GA MOF) enhances osteogenic/angiogenic gene expression and reduces inflammation [49,51]. Due to the risk of cytotoxicity arising from high ion concentrations, the release kinetics and dose need to be precisely regulated. The strength of metal–ligand bonds determines the structural stability of MOFs and has a key effect on ion release rates, which can be triggered by environmental factors, like pH, temperature, and ROS, triggering framework disintegration. The optimal balance between stability and controlled degradation ensures safe and effective therapeutic applications; for example, MOFs, including ZIF-8, ZIF-67, Mg-MOF-74, and HKUST-1, have been widely employed as ion donors [52]. However, as sustained release agents, most MOFs lack sufficient long-term biocompatibility and component synergy, which are challenges hindering their clinical translation.

### 2.3. Nanozymes

Nanozymes are artificial enzymes constructed based on nanomaterials to mimic the high catalytic efficiency properties of natural enzymes. In many catalytic fields, they are considered alternatives to natural enzymes due to their low cost, high stability, and excellent tolerance [53]. In recent decades, MOFs have also been widely used to prepare enzyme mimics. MOF nanozymes can be divided into two categories, according to their material composition. The first is the MOFs with catalytic activity, including two-dimensional (2D) MOF nanozymes and three-dimensional MOF nanozymes. For example, a 2D metal–organic skeleton-based nanozyme (Cu-TCPP(Fe)MOFs) was fabricated by coordinating between Cu(II) ions and peripheralcarboxyl groups of Fe(III) ion-centered metalloporphyrin to catalyze the decomposition of H_2_O_2_ [54]. The other class is composite nanozymes, which can be fabricated by combining MOFs and other functional materials to achieve the desired properties [55]. For example, Pt nanoparticles (Pt) have been loaded on the surface of MOFs based on Fe porphyrin and Zr^4+^ ions (PMOF(Fe)) to form Pt@PMOF(Fe), which has excellent catalase- and peroxidase-like activities [56]. The customizable structure provides MOFs with a variety of catalytic applications, and MOF-based nanozymes exhibit superior catalytic efficiency due to their high specific surface area and numerous binding sites. In addition, the robust synthetic framework exhibits greater stability against physicochemical degradation during transport to minimize loss of activity. These characteristics make MOF nanozymes a viable alternative to bioderived enzymes in catalytic applications [57]. Bio-MOF-1@AHTs, PEEK@ZIF-8(CEL), and UiO-66-modified titanium scaffolds enhance osteogenic gene expression (COL1, OPN, and BMP2), promote bone marrow mesenchymal stem cell (BMSCs) proliferation, and stimulate angiogenesis for bone integration [58,59,60]. For bone therapy, nanozymes based on MOFs focus on bone regeneration and OA treatment [61,62]. Although, nanocatalytic approaches based on MOFs offer a promising strategy for inflammatory disease treatment, among other things, translational studies are needed to evaluate their metabolic toxicity, long-term biocompatibility, immune responses, scalability, and so forth.

### 2.4. Phototherapeutic Agents

Phototherapy, including PDT and photothermal therapy (PTT), is a new and promising anticancer and antibacterial therapy with outstanding therapeutic effects and minor side effects on target tissues [63,64]. A specific light wavelength is used to activate the phototherapy agent when it is injected into the patient and selectively distributed in the target tissue to achieve the desired concentration, PDT produces ROS to interact with tissues, and PTT generates heat to increase the temperature; these are the most common mechanisms of phototherapy [65]. Therefore, the design and preparation of ideal photoactivatable agents are crucial for phototherapy, as photosensitizers (PSs) and photothermal agents (PTAs) are key components for PDT and PTT, respectively. MOFs with tunable structures have emerged as promising platforms for phototherapy [66]. The most common strategies for constructing MOF-based phototherapy agents are as follows: (1) PSs or PTAs are directly used as organic ligands to coordinate with metallic ions and clusters to fabricate MOF material. Porphyrins and their variations (such as TCPP, H_2_DBP, and TBBC) serve as photo-function groups and have been widely employed as organic ligands, especially for MOF-based PDT [63]. (2) Composite materials serve as phototherapeutic agents fabricated by PSs or PTAs and non-photo-function MOFs. This method allows for a greater variety of PSs and PTAs, in addition to porphyrins and their analogs. MOFs can be used as containers to load/encapsulate PSs or PTAs through non-covalent interactions; in addition, PSs and PTAs can be decorated om MOFs surfaces through covalent bonds [67,68]. For bone therapy, phototherapeutic agents based on MOFs exhibit antibacterial and osteogenic effects through mechanisms such as reactive oxygen species (ROS) generation, controlled ion release (e.g., Zn^2+^ and Cu^2+^), and the upregulation of genes (e.g., Runx2, ALP, Col I, and OCN) linked to bone formation [69,70,71]. As a new platform for phototherapeutic agents, MOFs not only inhibits the self-aggregation of photoresponsive molecules but also improves the efficiency of photoconversion. However, controlling selective light transmitted to organisms to trigger phototherapeutic agents remains challenging, as preventing light damage to normal tissues and guiding MOF aggregation can be difficult (Table 1).

## 3. Synthesis Technology for MOF Composites in Bone Disease

**Figure 2:**
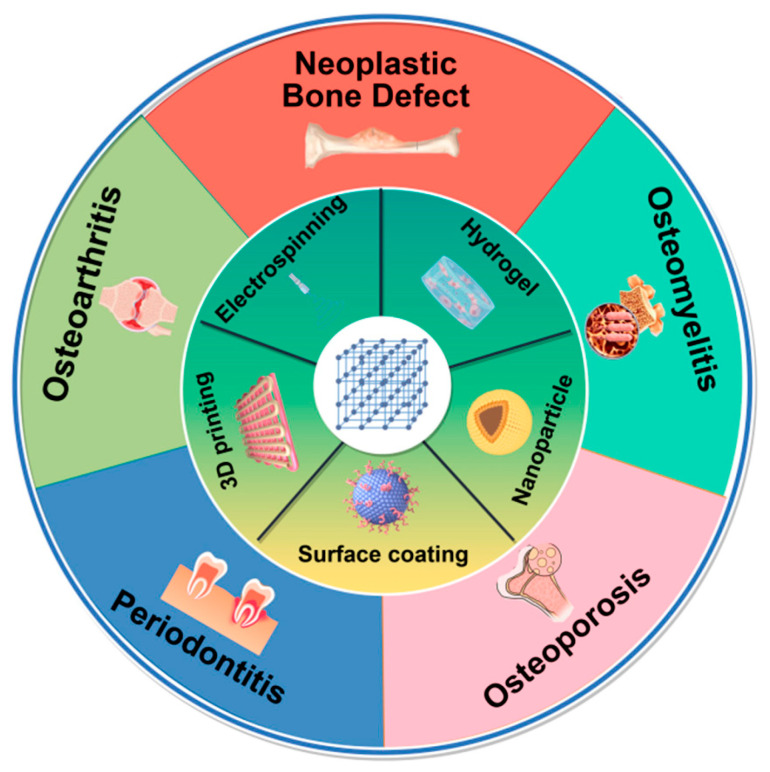
The synthesis technology for MOF composites in bone defect repair of bone diseases, including surface coating, electrospinning, 3D printing, hydrogels, and nanoparticles. The picture is made by Figdraw.

At present, reconstructing large bone defects caused by infection, trauma, and tumor resection remains a significant clinical challenge [9]. Using biomaterials to simulate natural bone structures with similar mechanical and biological properties is a common strategy for bone defect repair [81]. However, considering the poor bone conduction and induction performance of the material, MOFs can be mixed with the substrates through various forms—such as surface coating, electrospinning, 3D printing, and other methods—to exert excellent bone defect repair performance in bone diseases, including OA, osteoporosis, bone tumors, periodontitis, and osteomyelitis (Figure 2).

### 3.1. Surface Coating Materials

ZIF-8 can be coated on the surface of biphase calcium phosphate ceramics (Osteon II) to enhance bone induction performance. ZIF8-Osteon improves the adhesion and growth of hADSC cells, while also increasing early and osteogenic markers, such as Col-1, OCN, and SPP1 to promote bone regeneration [82]. A novel bone integration-promoting implant was prepared by modifying bio-MOF-1 on the surface of alkali-treated titanium (Bio-MOF-1@AHTs). This material has good biocompatibility and osteogenic properties, can promote the ALP activity of BMSCs, and improve osteogenic and mineralization by upregulating osteogenic genes (COL1, OPN, and BMP2) [58]. Ti was coated with probiotic-derived membrane vesicles (OMVs), tinidazole, and Cu-TCPP nanosheets to obtain a composite material for antibacterial and osteogenic promotion (CuTOT-Ti). It can promote the osteogenic differentiation of MC3T3-E1 cells and the polarization of M1-to-M2 macrophages. In the bone defect model, ultrasound activation can inhibit *S. aureus* and promote bone formation, mineralization, and angiogenesis [83]. Using the photocatalytic properties of ZIF-8, iodine was loaded onto ZIF-8 and coated on a micro-arc-oxidized (MAO) titanium base surface for antibacterial and osteogenic applications. Under near-infrared (NIR) irradiation, MAO + ZI significantly increased reactive oxygen species (ROS) levels of the bacteria and reduced the number of bacterial colonies by 95.48%. It also promoted osteogenesis by upregulating osteogenic genes (Runx2, ALP, Col I, and OCN) in hBMSCs [84]. In addition, coating zinc-based implants with a Cu-Mg MOF promotes bone integration and angiogenesis. The implant can release Zn^2+^, Mg^2+^, and Cu^2+^; promote the osteogenesis of MC3T3-E1 cells; and increase the expression of osteogenic genes (COL-1, ALP, Runx-2, and OCN). It can also promote HUVECs proliferation and migration and the expression of angiogenesis-related genes (eNOS and VEGF). In addition, this implant can activate NO production to destroy the bacterial membrane, showing strong antibacterial activity against *S. aureus* and *E. coli* [49].

Polyetheretherketone (PEEK) has good biocompatibility and similar mechanical properties to bone tissue. However, due to bioinertness and surface hydrophobicity, it requires surface modifications for bone repair applications [85]. Deng et al. loaded simvastatin (SIM), ZIF-8, and polydopamine (PDA) to construct a functionalized PEEK implant with near-infrared-responsive antimicrobial and osteogenic properties. Under 808 nm irradiation, SIM@ZIF-8-PDA accelerated the release of Zn^2+^, which had an obvious antibacterial effect on *Escherichia coli* (*E. coli*) and *Staphylococcus aureus* (*S. aureus*). In in vivo bone defect models, implants can effectively remove bacteria and promote bone regeneration [86]. Celecoxib (CEL) has been encapsulated in ZIF-8 and modified with DPA to PEEK to form a composite (PEEK@ZIF-8(CEL)). The implant promoted the proliferation of MC3T3-E1 cells and upregulated osteogenic genes to promote bone formation and mineralization [59].

### 3.2. Electrospinning Materials

MOFs can also be combined with other materials to form biofriendly bone-inducing materials through electrospinning. Zn-based MOF-derived nanocarbons (C-ZnO) can be mixed with poly(ε-caprolactone) (PCL) and form fiber scaffolds through electrospinning. This can promote the proliferation and osteogenic differentiation of MSCs, accompanied by the increased expression of laminin A/C and RUNX2 [87]. Toprak et al. encapsulated BMP-6 in ZIF-8 nanocrystals and incorporated them into PCL electrospun fibers, addressing BMP-6’s short half-life and localized efficacy. The PCL/BMP-6@ZIF-8 membrane enhances the proliferation, adhesion, and osteogenic differentiation of MC3T3-E1 cells. In vivo studies of rat skull defect models have shown that PCL/BMP-6@ZIF-8 significantly promotes bone regeneration [88].

Furthermore, mZIF-8/PLA has been prepared from ZIF-8 and polylactic acid (PLA) through electrospinning and simulated body fluid mineralization, showing good hydrophilicity and biocompatibility. The membrane can enhance the expression of osteogenic and vascularization genes in BMSCs, can promote M1-to-M2 polarization, and has good bone repair performance [51]. Exosomes secreted by human adipose-derived MSCs (hADSCs-Exos) can enhance the efficacy of bone regeneration biomaterials. The hADSCs-Exos has been encapsulated in poly(lactic-co-glycolic acid) (PLGA) and combined with a Mg^2+^-based organic framework and gallic acid to develop a scaffold (PLGA/Exo-Mg-GA MOF) through electrospinning. This scaffold has anti-inflammatory, osteogenic, and angiogenic properties. The release of Mg^2+^ can induce the expression of iNOS and COX-2 in RAW264.7 cells and inhibit proinflammatory mediators [48].

### 3.3. Hydrogel Materials

Hydrogel materials, such as gelatin methacrylate (GelMA) and methacrylate-modified hyaluronic acid (HAMA), can be coated with MOFs and other nanocomposites for bone defect repair. 2-ethylimidazole (elm)-doped ZIF-67 and GelMA have been photo-crosslinked in situ to form a hydrogel composite (GelMA@elm/ZIF-67) to achieve the controlled release of cobalt ions (Co^2+^). The release of Co^2+^ was sustained enough to correspond to the vascularization in the early stage of osteogenesis and increased the angiogenesis of the bone defect site by 17.03 ± 2.35%. It can also increase the activity of ALP and the expression of osteogenic gene in BMSCs to promote the formation of new bone [89]. ZIF-8 and dexamethasone (DEX) can be combined with wood aerogels to form DEX@ZIF-8, which can be used for skull defect repairs. The hydrogel enhances the adhesion and spread of rBMSCs and upregulates the expression of osteogenic genes (RUNX2, Col1A1) to promote osteogenic differentiation [90]. Moreover, ZIF-8-stabilized black phosphorus nanosheets (BPN) have been used to construct injectable nanocomposite hydrogels (GelMA/HAMA/BP@ZIF-8) by combining them with GelMA and HAMA. This hydrogel has good biocompatibility, can promote M1-to-M2 polarization of macrophages, and can reduce the expression of proinflammatory factors. Under NIR irradiation, GelMA/HAMA/BP@ZIF-8 has antibacterial and bone-forming capabilities, and it promotes late osteogenic differentiation and mineralization through the PI3K-Akt and calcium signaling pathways [30]. Metformin (Met) can be combined with ZIF-8 in GelMA to form a hydrogel for diabetic bone defects (GelMA/Met@ZIF-8) that simultaneously releases Met and Zn^2+^. At the same time, it promotes the transformation of M1 macrophages into M2 macrophages, reduces excess ROS production, maintains mitochondrial homeostasis, and thus, alleviates diabetic bone defects [31].

In tendon–bone regeneration, MOFs bind to cytokines and targeted peptides for the effective integration of tendon–bone interfaces. (DOPA)_4_-PEG_5_-N_3_ and DBCO-BMP-2 peptide have been used to construct a multifunctional hydrogel scaffold with GelMA (GM@MOF/DOPA-BMP-2) through a bioorthogonal click reaction. This scaffold can stimulate angiogenesis; can promote the osteogenic differentiation of BMSCs and the phenotype transformation of macrophages from M1 to M2; and has potential application value in tendon–bone reconstruction [91]. Ma et al. developed a double-targeted hydrogel scaffold for tendon–bone interface regeneration (LZIF-8/WMg-MOF@GEL) by combining tendon-targeting peptide-modified ZIF-8 and cartilage-targeting peptide-modified Mg-MOF into a GelMA matrix via UV polymerization crosslinking technology. The scaffold has sustained drug release, tissue targeting, and excellent tendon fibroblast biocompatibility, promoting tendon and bone tissue repair [92].

### 3.4. Three-Dimensional (3D) Printing Materials

Three-dimensional scaffolds with artificially controllable microstructures can bridge the complex morphology of bone defect edges, providing a potential strategy for bone tissue regeneration [93]. Extrusion-based 3D printing technology integrates ZIF-8 into a dicalcium phosphate dihydrate (DCPD) and PCL composite scaffold for bone regeneration. This scaffold has high-strength and a highly interconnected porous structure that sustainably releases Ca^2+^ and Zn^2+^, promoting the growth of BMSCs and new bone formation [94]. Modifying 3D-printed titanium scaffolds with UiO-66 can promote the optimal adhesion of BMSCs and HUVEC. Through the upregulation of osteogenic genes in BMSCs and enhancing the secretion of angiogenic factors in HUVEC, bone formation and angiogenesis can be promoted [60]. Furthermore, 3D-printed tricalcium β-phosphate (β-TCP) modified with Zn/Co-MOF can treat subchondral bone defects induced by osteoarthritis (OA). This scaffold has anti-inflammatory, antioxidant, and ROS scavenging capabilities that can promote the integration of subchondral bone defects [50].

### 3.5. Nanoparticle (NP) Materials

As ZIF-8 has a simple synthesis strategy, a strong loading capacity, and pH response degradation characteristics, it is widely used as an NP for drug and nucleic acid delivery in cells and in vivo [95]. Using the targeting properties of SCMs, ZIF-8 has been encapsulated in SCMs to enhance targeting and osteogenic regeneration. SCM/ZIF-8 can regulate the sustained release of Zn^2+^ and promote osteogenic differentiation and mineralization by upregulating the expression of OPN and OCN. In in vivo femur defect models, the material has good biocompatibility and promotes the formation of new bone tissue [74]. Furthermore, ZIF-8 loaded with dexamethasone (DEX) has been coated on SCMs to construct a bionic material for targeted bone repair (DEX@ZIF-8-SCM). These NPs can promote MSC growth, ALP activity, and OCN expression. Bone formation is accelerated through the phosphoinositol 3-kinase (PI3K)-AKT signaling pathway, thereby promoting bone tissue repair [96]. In addition, bimetallic MOF NPs (Pt@ZIF-8@La) have been constructed by encapsulating Pt nanozyme and lanthanum (La) with ZIF-8. These NPs have high active oxygen scavenging capacity, reduce cellular inflammation, and promote osteogenic differentiation and bone regeneration by upregulating the osteopontin (OPG)–nuclear factor-κB ligand (RANKL) ratio [61].

## 4. Applications of MOFs in Bone Disease Therapy

### 4.1. MOFs in Osteoarthritis (OA)

OA is a degenerative joint disease with pathological features, including cartilage destruction, subchondral bone remodeling, and synovitis. Approximately 250 million people worldwide currently suffer from OA, creating a significant social and economic burden [97,98]. MOF-based biomaterials based on tissue targeting and controlled slow release are becoming an important method for OA therapy [99] (Table 2).

The acidic substances produced by chondrocytes and synovial cells in the OA microenvironment make the pH lower than the normal physiological state. The acid reaction degradation properties of MOF materials, such as MIL-100(Fe) and ZIF-8 make them good carriers for sustained drug release [100,101]. Xiong et al. modified pH-responsive MIL-100(Fe) with hyaluronic acid (HA) and protocatechuic acid (PCA) (MOF@HA@PCA) to treat OA. These NPs have good biocompatibility, reduce IL-1β-induced chondrocyte inflammation and the expression of matrix-degrading enzymes (MMP1, MMP3, and MMP13), and promote Col2a1 and Acan expression. Cartilage degeneration can be alleviated by inhibiting the expression of MMP-13 in the cartilage tissue of OA rats [62]. Some studies have used ZIF-8 as a pH response vector. Quercetin, with anti-inflammatory and antiapoptotic properties, is delivered to chondrocytes via ZIF-8 (Qu@ZIF-8), which enhances chondrocyte autophagy by inhibiting the PI3K/Akt signaling pathway, promotes the expression of cartilage anabolic genes, and protects cartilage integrity [102]. MIL-101-NH_2_ combines with curcumin (CCM) and siHIF-2α to form pH-responsive NPs with a 59.7% controlled release effect under acidic conditions. These NPs have good biocompatibility and inhibit hypoxia-induced cartilage dysfunction in vivo [103]. In rheumatoid arthritis (RA), the CRIg-CD59 complement is encapsulated by ZIF-8 nanoparticles and forms a ZIF8@CRIg-CD59@HA@ZA complex with hydroxyapatite (HA) and zoledronic acid (ZA). In an acidic environment, the platform delivers CRIg-CD59 to the affected site of RA and can inhibit complement activation and repair VSIg^4+^ macrophage barrier function, while targeting osteoclasts to inhibit their differentiation [32]. This acid-sensitive property endows MOFs with excellent drug-sustained release performance, allowing them to be induced for release in acidic microenvironments.

Macrophages play an important role in OA. M1 macrophages are involved in tissue fibrosis, and M2 macrophages promote cartilage repair and alleviate arthritis [104]. Sun et al. developed a nanogel (KZIF@HA) for the sustained release of Kartogenin (KGN). Compared with KGN, KZIF@HA can increase cartilage tissue permeability by 40%. This nanogel promotes the polarization of M2 macrophages and the secretion of IL-10 and then inhibits JNK and ERK pathways in chondrocytes to promote chondrocyte anabolism and increase the secretion of type II collagen [105]. Folic acid (FA)-modified UiO-66 can target baicalin to M1 macrophages, effectively reducing ROS and promoting the polarization of M2 macrophages, thus alleviating synovial hyperplasia caused by OA [106]. These studies provide promising evidence for drug-loaded MOF nanoparticles in the treatment of OA by regulating the M1-M2 phenotypic transformation.

In addition, Liu et al. modified MOFs using biomimetic lubrication materials and developed a series of NPs with photothermal response and lubrication properties in OA treatment. The bionic lubrication nanosystem ZIF-8-PDA-HA was formed by combining HA and dopamine, which can load diclofenac sodium (DS) up to 99%. Under infrared irradiation, the lubrication system can reduce the friction coefficient indicated by wear, slow release DS, and enhance the expression of Col2α and Acan in chondrocytes [107]. Poly(ethylene glycol)-graft-poly(N-isopropylacrylamide) (PEG-g-PNIPAm) copolymer coated with MIL-101(Cr) forms a nanogel mixing system with a thermal response and lubrication properties. The expression of Sox-9, Col2α1, and aggrecan can be upregulated in TNF-α-induced chondrocytes, while the expression of MMP1, IL-6, and COX-2 can be downregulated, and ECM degradation can be inhibited [108]. Furthermore, by coating P(NIPAm-g-PEGMax) copolymer microgel on the surface of MIL-101(Cr), another heat-responsive drug-sustained release system was developed with good lubrication properties and OA therapeutic potential [109]. In addition to components with biological regulatory functions, combining materials with lubricating properties and MOFs can synergistically promote cartilage tissue regeneration in the in vivo environment.

Nanozyme is a kind of artificial enzyme with multi-enzyme activity that has the advantages of good biocompatibility, low cost, easy synthesis, etc. It has been widely applied in OA treatment [110]. Zhang et al. loaded Mn_3_O_4_ nanoparticles into UIO66 using the hydrothermal method and modified with mitochondrial-targeted triphenylphosphine (TPP) groups. Mn_3_O_4_/UIO-TPP clears mitochondrial ROS through the subcellular barrier, inhibits chondrocyte apoptosis and MMP13 expression, and effectively alleviates OA in rats [111]. The Cu-based Cu MOF nanozyme has better ROS clearance abilities than CuO and copper monoatomic nanozyme; it can promote the polarization of the macrophage M1 subtype into the M2 subtype, downregulate MMP13, and inhibit the degradation of cartilage matrix [112]. The ZIF-8 delivery platform (miR/IrO_2_@ZIF-8), loaded with IrO_2_ NPs and antisense oligonucleotide, can escape from lysosomes and penetrate human cartilage tissue to a depth of 1.5 mm, effectively reducing the expression of IL-6 and ADAMTS-5 in chondrocytes. The integrity of cartilage ECM can be protected by upregulating the expression of COLII and ACAN [110]. Antioxidant activity plays a key role in maintaining joint homeostasis. MOF materials can not only regulate ROS by loading nanozymes but also exert antioxidant capacity through specific metal atom sites and have great potential in alleviating the oxidative stress of OA.

**Table 2:** Summary of the application of MOF-based composite materials for the treatment of osteoarthritis.

MOF Composites	Form of the Composites	Primary Role of MOFs	Loaded Drugs and Loading Efficiency	Metal Ions/Clusters	Cellular Biological Functions	Mechanism	In Vivo Effects	Refs
MIL-100(Fe)@HA@PCA	Nanoparticle	Drug carrier	Hyaluronic acid (~21.6%); protocatechuic acid (~19.4%)	Fe^3+^	Promoting chondrocyte proliferation; relieving inflammation	/	Promoting cartilage regeneration: inhibit MMP-13 expression	[62]
Qu@ZIF-8	Nanoparticle	Drug carrier	Quercetin (~23%)	Zn^2+^	Promoting cartilage anabolism; anti-inflammatory; inhibiting IL-1β-induced apoptosis	Inhibiting PI3K/Akt signaling pathway	Maintaining cartilage structure integrity and glycosaminoglycan synthesis	[102]
MIL-101-NH_2_	Nanoparticle	Drug carrier	Curcumin (~25.9%); siHIF-2α; hyaluronic acid	Fe^3+^	Promoting cartilage anabolism; inhibiting inflammatory factors	/	Promoting cartilage anabolism; inhibiting inflammatory factors	[103]
ZIF8@CRIg-CD59@HA@ZA	Nanoparticle	Drug carrier	CRIg-CD59 (>90%); Zoledronic acid (~73.6%)	Zn^2+^	Inhibiting bone resorption; repairing VSIg^4+^ macrophage barrier	/	Reducing synovial hyperplasia; protection of articular bone	[32]
KZIF@HA	Nanoparticle	Drug carrier	Kartogenin; hyaluronic acid	Zn^2+^	Promoting cartilage anabolism; promoting M1-to-M2 macrophage polarization	Inhibiting JNK and ERK pathways in chondrocytes	Cartilage protection; inhibiting joint inflammation	[105]
Bai@FA-UIO-66-NH_2_	Nanoparticle	Drug carrier	Baicalin	Zr^4+^	Reducting ROS; promoting M1-to-M2 macrophage polarization	Modulation of immune homeostasis	Cartilage protection; reducing bone hyperplasia; recover subchondral bone structure	[106]
ZIF-8-PDA-HA	Nanoparticle	Drug carrier	Diclofenac sodium (~99%)	Zn^2+^	Promoting cell proliferation; accelerated lubrication	/	/	[107]
MIL-101(Cr)@PEG-g-PNIPAm	Hydrogel	Drug carrier	Diclofenac sodium (~29.2%)	Cr^3+^	Promoting cartilage anabolism; anti-inflammatory; accelerating lubrication	/	/	[108]
MIL-101(Cr)@P(NIPAm-gPEGMa_x_)	Hydrogel	Drug carrier	Diclofenac sodium (~23.8%)	Cr^3+^	Anti-inflammatory; accelerating lubrication	/	/	[109]
Mn_3_O_4_/UIO-TPP	Nanoparticle	Nanozyme	/	Mn_3_O_4_; Zr^4+^	reducting mitochondrial ROS; inhibiting oxidative and inflammatory	/	Cartilage protection; inhibiting IL-6 and MMP-13 expression	[111]
Cu MOF	Nanoparticle	Nanozyme	/	Cu^2+^	Reducting ROS; promoting M1-to-M2 macrophage polarization	/	Inhibiting ECM degradation; improving hypoxia; inhibiting synovitis	[112]
miR/IrO_2_@ZIF-8	Nanoparticle	Nanozyme	AntagomiR-181a	Zn^2+^	Reducting ROS; relieving inflammation; promoting cartilage anabolism;	/	Inhibiting ECM degradation; reducing bone hyperplasia; recover subchondral bone structure; inhibiting MMP-13 and ADAMTS-5 expression	[110]

### 4.2. MOFs in Neoplastic Bone Defects

#### 4.2.1. Breast Cancer-Induced Osteolysis

Bone metastasis in breast cancer is the main cause of death for this disease. Breast tumor cells promote osteoclast differentiation and osteolysis through IL-8 and other factors on the bone surface [113]. The release of nutrients from the bone matrix promotes the survival and invasion of breast tumor cells [114]. Inhibiting tumor cells and osteoclasts is an important strategy for treating breast cancer-induced osteolysis (Table 3).

UiO-66 NPs were combined with immunostimulatory cytosine–phosphate–guanosine(CpG) and ZA to form bone-targeting NPs (BT-isMOF). The system can strongly bind with calcium phosphate in bone tissue and inhibit osteoclast formation. At the same time, it can promote the polarization of M1 macrophages and the expression of pro-inflammatory factors and inhibit breast tumors. In vivo experiments show that BT-isMOF can effectively alleviate bone resorption caused by breast cancer bone metastasis [115]. Ge et al., utilizing the bone-targeting properties of ZA, simultaneously loaded the anticancer drug 5-fluorouracil (5-Fu) and the organic photothermal molecule indocyanine green (ICG) into ZIF-90 to develop a photothermal responsive bone-targeted drug delivery vector (5-Fu/ICG@ZIF-90-PEG-ZOL). Under NIR light, the NPs induced the apoptosis of MCF-7 cells by inhibiting DNA replication. NPs can reduce tumor volume and have no organ toxicity, showing an inhibitory effect on breast tumors in vivo [116]. Further, a bone-targeted ICG@Cu_2_-xSe-ZIF-8 composite was developed by combining Cu_2_-xSe chemodynamic therapy (CDT) with PTT to inhibit tumor growth and protect bone tissue. Under 808 nm laser irradiation, the complex can effectively inhibit the proliferation and invasion of MDA-MB-231 in vitro and inhibit osteoclastic differentiation. In a mouse model of breast cancer bone metastasis, NPs inhibit tumor growth and tumor cell-induced osteolysis [117]. Taking advantage of the PTT and CTT properties of MOF materials—combined with tissue-targeted peptides and drugs to precisely regulate the tumor site—it is possible to kill tumors while inhibiting osteolysis. This is a key advantage of MOF materials in the treatment of bone defects caused by tumors.

#### 4.2.2. Osteosarcoma (OS)

OS is a malignant tumor that occurs in the bone, most commonly in children or adolescents, leading to 20,700 deaths in China every year [118]. Existing treatments for OS mainly include chemotherapy and surgery, usually requiring high doses of radiation for tumor ablation. Hypoxia is one of the reasons for the poor radiotherapy effect of osteosarcoma [119]. Antitumor NPs (HA@MOF/D-Arg) loaded with MIL-100(Fe), D-arginine (D-Arg), and HA can sensitize tumor cells to radiation. Under hypoxia conditions, NPs can enhance the production of free radicals in osteosarcoma (OS) cells, promote tumor ablation, and inhibit lung metastasis [120]. NPs formed by D-Arg with the chemotherapy drug telaprazine (TPZ) and Fe-based MOFs can catalyze the formation of NO from H_2_O_2_ to alleviate the hypoxia in OS cells and kill them by inducing DNA damage under X-ray irradiation [121]. Ge et al. developed a pH-responsive antitumor NPs (CUR-BMS1166@ZIF-8@PEG-FA, CBZP) by loading the immune checkpoint inhibitor BMS1166 and the autophagy activator curcumin (CUR) into ZIF-8. At pH = 6.5, CUR induced the expression of autophagy-associated protein ATG5 in OS cells and promoted HMGB1 release and calmodulin exposure, thus inducing autophagy dependent immunogenic cell death (ICD). In addition, the NPs inhibited the PD-1/PD-L1 axis by releasing BMS1166, forming a synergistic antitumor effect [122].

In addition to inhibiting OS formation, MOF composites can also simultaneously promote osteogenic differentiation and vascularization and inhibit osteolysis; they can jointly repair bone defects caused by OS. In terms of osteogenic differentiation, chitosan/NH_2_-MIL-125(Ti) scaffolds can be loaded with doxorubicin (DOX) to eliminate OS. Its superior biocompatibility and specific surface area can also enhance the adhesion, alkaline phosphatase (ALP) activity, and osteogenic differentiation of MC3T3-E1 cells [123]. UiO-66 NPs loaded with DOX (DOX@UiO-66-NH_2_) can release DOX in acidic environments, blocking nucleic acid synthesis and OS cell growth. They can also regulate the PI3K-Akt or MAPK signaling pathway to promote osteogenic differentiation [33]. A 3D-printed scaffold with Cu icons and MOFs can be used for OS treatment and bone repair. The scaffold not only eliminates tumor cells by generating reactive oxygen species (ROS) through the photothermal effect and Fenton reaction but also promotes osteogenic differentiation by upregulating the expression of COL1, OCN, BMP-2, and RUNX2 in hBMSCs [69]. As angiogenesis plays an important role in bone repair, a composite scaffold (CU-TCPP-TCP) was prepared by adding ultra-thin Cu-TCPP MOF nanosheets to a β-tricalcium phosphate (β-TCP) ceramic scaffold. Under NIR irradiation, the scaffold can eliminate residual OS cells and promote the osteogenesis and angiogenesis of hBMSCs and HUVECs, respectively, which jointly enhance new bone growth in vivo [124]. Qu et al. fabricated a photothermally responsive injectable multifunctional implant (Co-TCPP/CPC) by incorporating cobalt coordinated tetrakis (4-carboxyphenyl) porphyrin (Co-TCPP) into calcium phosphate cement (CPC). Under photothermal stimulation, OS cells can be ablated by inducing protein denaturation, and the expression of osteogenic and angiogenic factors can be up-regulated to promote bone defect repair [70]. In terms of inhibiting osteolysis in OS, Liu et al. developed a biomimetic multifunctional nanoplatform (PDA-MOF-E-M) that encapsulates Fe-MOF and erastin within the cell membrane of OS to achieve targeted therapy. This platform can induce ferroptosis in OS cells by promoting lipid peroxide accumulation. Beyond that, this platform inhibited NFATc1 expression and promoted TRAF3 expression, thereby inhibiting osteoclast differentiation and osteolysis under NIR irradiation [125]. Another nano-drug delivery system (V-RZCD) modified with vascular endothelial growth factor (VEGF) ligands can be targeted to treat OS and osteolysis. V-RZCD promotes the apoptosis of OS cells by downregulating Bcl-2 and increasing the expression of caspase-3. Furthermore, it inhibits osteoclast differentiation by regulating the RANKL/RANK/OPG signaling pathway, thereby protecting bone tissue structure [126]. Taken together, in the design of MOF composite materials for osteosarcoma treatment, in addition to considering the elimination of OS cells, multiple dimensions must be considered, such as promoting osteogenic differentiation, promoting vascularization, and inhibiting osteoclastic differentiation. These functions can endow MOFs with a synergistic effect in repairing of bone defects in OS.

**Table 3:** Summary of the application of MOF-based composite materials for the treatment of neoplastic bone defects.

Neoplastic Bone Defects	MOF Composites	Form of the Composites	Primary Role of MOFs	Loaded Drugs	Metal Ions/Clusters	Cellular Biological Functions	Mechanism	In Vivo Effects	Refs
Breast Cancer	BT-isUiO-66	Nanoparticle	Drug carrier	Zoledronic acid (~3.14 wt%)	Zr^4+^	Reducting ROS; inhibiting bone resorption; promoting M1 macrophage polarization	Activating TLR9 signaling transduction	Inhibiting tumor; protection of tibial tissue structure	[115]
	5-Fu/ICG@ZIF-90-PEG-ZOL	Nanoparticle	Drug carrier	5-fluorouracil; zoledronic acid (~23.13%)	Zn^2+^	Inducing apoptosis of MCF-7 cells under NIR	Inhibiting DNA replication	Bone-targeted; inhibiting tumor	[116]
	ICG@Cu_2_-xSe-ZIF-8	Nanoparticle	Photosensitizer	/	Cu_2_-xSe	Promoting tumor cell apoptosis; inhibiting bone resorption	Inhibiting p65 and NFATc1 pathways	Inhibiting tumor; inhibiting cancer cells-induced osteolysis	[117]
Osteosarcoma	HA@MOF/D-Arg	Nanoparticle	Drug carrier	D-arginine	Fe^3+^	Promoting tumor cell apoptosis	Promoting DNA damage	Enhancing tumor ablation; preventing lung metastasis; alleviating tissue hypoxia	[120]
	PDA-cloaked Fe-MOF	Nanoparticle	Drug carrier	D-arginine; tirapazamine	Fe^3+^	Promoting tumor cell apoptosis	Producing ROS	Enhancing tumor ablation; preventing lung metastasis	[121]
	CUR-BMS1166@ZIF-8@PEG-FA	Nanoparticle	Drug carrier	Curcumin; BMS1166	Zn^2+^	Inducing immunogenic cell death	Activating autophagy; Inhibiting PD-1/PD-L1 signaling	Inhibiting tumor growth	[122]
	NH_2_-MIL-125(Ti)	Scaffold	Drug carrier	Doxorubicin	Ti^4+^	Promoting tumor cell apoptosis; promoting osteogenesis	/	Inhibiting tumor growth; promoting bone repair	[123]
	DOX@UiO-66-NH_2_	Nanoparticle	Drug carrier	Doxorubicin	Zr^4+^	Promoting tumor cell apoptosis; promoting osteogenesis	Promoting osteoblast differentiation via activating PI3K-Akt and MAPK signaling pathways	Inhibiting tumor growth	[33]
	PCL@Cu-HHTP	Scaffold	Photosensitizer	/	Cu^2+^	Promoting tumor cell apoptosis; promoting osteogenesis	/	Inhibiting tumor growth	[69]
	Cu-TCPP-TCP	Scaffold	Photosensitizer	/	Cu^2+^	Promoting tumor cell apoptosis; promoting osteogenesis; promoting vascularization	/	Inhibiting tumor growth; promoting bone repair	[124]
	Co-TCPP/CPC	Scaffold	Photosensitizer	/	Co^2+^	Promoting tumor cell apoptosis; promoting osteogenesis; promoting vascularization	/	Inhibiting tumor growth; promoting bone repair	[70]
	PDA-MOF-E-M	Nanoparticle	Photosensitizer	Erastin	Fe^3+^	Promoting tumor cell apoptosis; inhibiting osteoclastic differentiation	/	Inhibiting tumor growth; inhibiting osteolysis	[125]
	V-RZCD	Nanoparticle	Drug carrier	Doxorubicin; zoledronic acid	Ca^2+^	Promoting tumor cell apoptosis	/	Inhibiting tumor growth; inhibiting osteolysis	[126]

### 4.3. MOFs in Osteoporosis

Osteoporosis is a chronic skeletal disorder marked by reduced bone density, bone microarchitecture deterioration, heightened bone fragility, and susceptibility to fractures [127]. Osteoporosis affects over 200 million individuals globally, leading to significant morbidity characterized by debilitating fractures and substantial physical limitations that impair quality of life [128]. In inflammatory, estrogenic, and age-related osteoporosis, imbalances in the expression of RANKL and OPG in the bone microenvironment lead to impaired osteoblast function and enhanced osteoclast formation, ultimately disrupting the balance of bone remodeling processes [129].

The release of active ingredients from biomaterials to support apatite formation is an important step in bone regeneration. Biocompatible MOFs composed of Sr^2+^ and Ca^2+^ can simulate the dissolution rate of these ions in body fluids and have good biocompatibility [130]. A complex of self-sacrificing MOFs with metal ions that promote osteoblast activity and bisphosphonate (BP) drugs that inhibit osteoclast activity can achieve controlled release for up to 20 days. For non-BP drugs, etidronate (ETID) and alendronate are released faster, while NER and PAM are released the slowest. In the case of compound BP drugs and metal ions, the sustained release effect of Ca-ETID is the best [131]. Hou et al. developed five Ca^2+^-based zwitterionic dicarboxylate MOFs to address osteoporosis. These bioMOFs can promote the expression of bone formation markers, such as ALP and Runx2 and promote osteogenic differentiation. In OVX mice, MOFs can increase serum OCN levels, promote collagen deposition, and improve bone microstructure, thus preventing bone loss [132]. Therefore, in the treatment of osteoporosis, the ionic composition of MOFs designed to release based on bone tissue composition plays an important role in bone regeneration.

In trauma and aging pathology, exposing MSCs to ROS results in cell senescence and weakened osteogenic differentiation, leading to osteoporosis [133]. Zhang et al. developed novel titanium–organic framework implants (AHT-Ce/Sr MOF) coated with Ce and Sr ions as bioactive molecules, which can be used for ROS regulation in MSCs. AHT-Ce/Sr MOF can reduce ROS levels by activating AMPK in MSCs and improve mitochondrial division, fusion, and autophagy to reverse aging. In vivo implantation experiments show that AHT-Ce/Sr MOF can effectively promote bone mineral and bone regeneration [134]. To take advantage of the acidic environmental characteristics of osteoclasts, an acid-responsive MOF system, ZIF8-NaHCO_3_@Cas9 (ZNC), was designed by incorporating sodium bicarbonate (NaHCO_3_) and RANKL-CRISPR/Cas9 plasmids for osteoporosis treatment. The ZNC NPs effectively neutralize the acidic microenvironment, improving transfection efficiency and inhibiting the formation of osteoclasts. In osteoblasts, ZNC NPs can reduce ROS levels and delay cell senescence. In addition, ZNC promotes osteogenic differentiation and mineralization by downregulating RANKL expression. In OVX mouse models, ZNC can promote bone mass and inhibit osteoclast and senescent cell formation [135]. Although MOFs of different ligands can be involved in stem cell osteogenic differentiation, senescence, and osteoclast formation in OP, more detailed molecular mechanisms have not yet been clarified, necessitating further exploration (Table 4).

### 4.4. MOFs in Periodontitis

Oral diseases such as periodontitis attract tremendous attention all over the world, Periodontitis alone accounts for over 5.4 million cases, making it the sixth most prevalent disease in the world [136]. Periodontitis is a chronic inflammatory disease characterized by the destruction of periodontal tissue, including gums, periodontal ligaments, and alveolar bone that often leads to tooth loss if untreated. This disease is primarily triggered by anaerobic bacterial biofilms, such as *Porphyromonas gingivalis* (*P. gingivalis*), which results in immune responses that exacerbate tissue damage [8]. The current treatment is mainly mechanical debridement and antibiotic therapy. While the difficulty in reaching certain periodontal areas and increased microbial resistance render existing approaches almost ineffective [137]; innovative biomaterials, such as MOFs, show potential in addressing these challenges (Table 5).

Li et al. used ZIF-8 to encapsulate and deliver minocycline hydrochloride (Mino) to develop NPs with pH response properties. The NPs are effectively absorbed by human periodontal ligament cells (hPDLCs), and the release of Zn^2+^ and Mino reduces pro-inflammatory cytokines through the AKT/GSK3β/NRF2 pathway, which further reduces alveolar bone resorption and improves bone density in vivo [75]. ZIF-8 PDA formed a cell culture platform (PP/PDA/ZIF-8 membrane) on the surface of the polypropylene (PP) membrane with good wetness, roughness, and stiffness, promoting the proliferation of dental pulp stem cells (DPSCs). It also promotes osteogenic differentiation by regulating the expression of OPN and bone morphogenetic protein 2 (BMP2) [138]. Moreover, dexamethasone can be synthesized by combining ZIF-8 nanoparticles with polyphosphate methacrylate (PPEMA) and gelatin methacrylate (GelMA) to construct a nanohydrogel released in the acidic microenvironment of the periodontal pocket (DZIF@PGel). The continuous release of Zn^2+^ from ZIF-8 can destroy the bacterial biofilm and inhibit the growth of key periodontal pathogens. Hydrogels promote the proliferation of human gingival fibroblasts (HGFs) and BMSCs. They can also inhibit osteoclast activity in the inflammatory microenvironment and support bone regeneration in vivo [139]. These research results contribute to the development of more MOFs for regulating osteogenic and osteoclastic differentiation and bone remodeling, demonstrating the potential of MOFs in the effective treatment of periodontitis.

In addition, ZIF-8 loaded with miR-27a can be used as a coating material to release miR-27a and Zn^2+^ in a pH-responsive manner, reprograming the mitochondrial metabolism of macrophages to prevent peri-implant inflammation. L-MOF-agomir coating transforms RAW264.7 cells from the M1 phenotype into the M2 phenotype, a transition mediated by improved mitochondrial activity and a metabolic shift from glycolysis to oxidative phosphorylation, both of which are critical for reducing inflammation and promoting tissue repair. The supernatant secreted by macrophages also upregulates osteogenic markers, such as RUNX2 and ALP in BMSCs, indicating its potential for bone regeneration [140]. Quercetin can also be encapsulated in hydrogels with ZIF-8, and a five-in-one composite system has been developed (SFD/CS/ZIF-8@QCT). This system inhibits *Porphyromonas gingivalis* and demonstrates rapid and effective hemostatic capabilities, along with excellent blood compatibility. It reduces inflammatory factors by promoting M1-to-M2 macrophage polarization and immune reprogramming. By activating the PI3K/Akt/GSK-3β signaling pathway and inhibiting the production of ROS in PDLCs, it accelerates the periodontal repair process [141]. MOFs regulate macrophage inflammation, providing new ideas for the treatment of periodontitis. Through the regulation of the M1-M2 phenotypic transition, periodontal regeneration can be directly or indirectly affected.

In addition to ZIF-8, UiO-66 and CuTCPP can also be used as drug delivery carriers for periodontitis bone regeneration. UiO-66 is loaded with thymol and carvacrol to exert its antibacterial and anti-inflammatory properties. Car@UiO-66 and Thy@UiO-66 have good biocompatibility and enhance osteogenic activity and vascularization in hPDLCs. By inhibiting NF-κB signaling and reducing the expression of pro-inflammatory factors, macrophages can be polarized from the M1 phenotype into the M2 phenotype, showing a strong anti-inflammatory effect. In vivo experiments show that the composite hydrogel can promote the formation of new bone in alveolar bone defects and reduce inflammation [72]. Lam et al. designed hierarchical mesoporous zirconium-based MOF NPs (HMUiO-66-NH2) that slowly release zirconium ions (Zr^4+^) in acidic environments. These NPs break down hydrogen peroxide without producing harmful hydroxyl radicals, thereby reducing ROS levels. In addition, activating Wnt and TGF-β signaling pathways can enhance the osteogenic differentiation of BMSCs and protect bone structure defects caused by periodontitis [142]. In one study, CuTCPP modified by atomic-layer Fe_2_O_3_ was incorporated into the polyethylene glycol (PEG) matrix to form photoresponsive ointment (CuTCPP-Fe_2_O_3_), which can be used to treat periodontitis. CuTCPP-Fe_2_O_3_ exhibits strong antibacterial activity through ROS and ion release. In vivo studies have shown that this ointment reduces alveolar bone loss and tissue inflammation, promotes collagen deposition and angiogenesis, and alleviates periodontitis [73]. The MOF formed by Mg and gallic acid (GA) forms dynamic hydrogels with 4-formylphenylboronic acid, dextran, and carboxymethyl chitosan (CSBDX@MOF), which can deliver drugs in an acidic environment. The release of Mg^2+^ promotes osteogenic differentiation, while GA reduces oxidative stress and modulates the immune response by polarizing macrophages from M1 into M2. This hydrogel also demonstrates strong antibacterial action against *P. gingivalis* and *Actinobacillus actinomycetemcomitans*. In a rat model of periodontitis, CSBDX@MOF reduced alveolar bone loss and enhances bone regeneration and collagen deposition, which are key factors in tissue repair [143].

### 4.5. MOFs in Osteomyelitis

Osteomyelitis is a bacterial bone disease caused by trauma and joint replacement. If not treated in time, it will lead to sepsis, inflammation, and bone destruction [144]. Existing treatments consist mainly of long-term antibiotics and multiple debridement surgeries. However, due to antibiotic resistance and the pain caused by surgery, safer and more effective treatment options need to be considered [145]. Photodynamic therapy, photothermal therapy, and microwave therapy can effectively replace antibiotic-based therapies, have superior antibacterial and bone repair properties, and have become effective strategies for osteomyelitis treatment [71,146] (Table 6).

Among non-antibiotic antimicrobial therapies, sonodynamic therapy (SDT) has emerged as a reliable way to eliminate bacteria and repair tissue [147]. A zirconium-based porphyrin MOF (HNTM) is doped with Au and single atoms to provide it with SDT properties, and it is coated with a red blood cell (RBC) membrane to improve biocompatibility. The HNTM-Pt@Au system can effectively treat osteomyelitis induced by methicillin-resistant *Staphylococcus aureus* (MRSA); promote M1-to-M2 macrophage polarization by inhibiting iNOS expression and promoting TGF-β expression; and promote bone regeneration [148]. The piezoelectric material MoS_2_ forms a piezoelectric-enhanced SDT with HNTM and the RBC membrane, and its ROS and mechanical properties can effectively eliminate MRSA by promoting DNA damage, oxidative stress, tryptophan metabolism, and so on. In osteomyelitis rats, RBC-HNTM-MoS_2_ can relieve inflammation and promote the polarization of macrophage M1 into M2 [149]. Adding a sonosensitizer can effectively improve the antibacterial properties of photosensitive materials. HN-Ti_3_C_2_, consisting of HNTM and Ti_3_C_2_, can kill 99.75% of MRSA under low-intensity ultrasound (US) and promotes stem cell proliferation and osteogenic differentiation through the Wnt, calcium, and TGF-β signaling pathways. HN-Ti_3_C_2_ eliminates bacterial infection and promotes bone regeneration in a rat model of osteomyelitis [146]. Cerium-based MOF (CeTCPP) supported by Au NPs can also be used for SDT in osteomyelitis. Under US, Au NPs transform Ce^3+^ nodes into Ce^4+^ nodes and enhance ROS generation. Therefore, within 20 min of US, the antibacterial effect of CeTCPP-Au on *Staphylococcus aureus* and *Escherichia coli* can reach more than 99%, with good biocompatibility and deep antibacterial and anti-inflammatory effects in vivo [150]. Microwave (MV)-reactive materials are also an effective antibiotic-free treatment strategy. CNT-2D MOF formed by oxidized carbon nanotubes (CNT) and ultrasmall Cu-based 2D MOF can absorb microwaves and convert them into heat through heterojunction interface polarization. They can also destroy bacterial membranes through Cu ion release. This system showed antibacterial activity against seven pathogenic bacteria and good biocompatibility in vivo [151]. These methods of converting physical substances into biochemical signals through MOF complexes based on SDT and microwaves address the drawback that bone marrow infections require high-dose systemic antibiotic treatments, while also avoiding the limitations of antibiotic toxic side effects and bacterial resistance.

**Table 6:** Summary of the application of MOF-based composite materials for the treatment of osteomyelitis.

MOF Composites	Form of the Composites	Primary Role of MOFs	Metal Ions/Clusters	Cellular Biological Functions	Mechanism	In Vivo Effects	Refs
HNTM-Pt@Au	Nanoparticle	Sound sensitizer	Pt, Au	Antibacterial; Promoting osteogenesis and mineralization; promoting M1-to-M2 macrophage polarization	Inhibiting iNOS and promoting TGF-β in macrophages	Inhibiting MRSA-infected osteomyelitis; preventing bone destruction	[148]
HNTM-MoS_2_	Nanosheet	Sound sensitizer	MoS_2_	Antibacterial; promoting M1-to-M2 macrophage polarization	/	Eliminating bone infection; inhibiting inflammation; inhibiting bone loss	[149]
HN-Ti_3_C_2_	Nanosheet	Sound sensitizer	Ti_3_C_2_	Antibacterial; Promoting osteogenesis and mineralization	Activating calcium, MAPK, and Wnt signaling pathways	Eliminating bone infection; inhibiting bone loss	[146]
CeTCPP-Au	Nanoparticle	Sound sensitizer	Au	Antibacterial	/	Eliminating bone infection; relieving inflammation	[150]
CNT-CuHHTP	Nanotube	Microwave dynamic therapy	Cu^2+^	Antibacterial	/	Eliminating bone infection; relieving inflammation	[151]

## 5. Conclusions and Prospects

With the development of tissue engineering technology, the use of functional MOF composites as emerging biomaterials for the treatment of bone diseases has become a potential trend in biomedical applications. MOFs can function as drug carriers, metal ion donors, nanozymes, and photosensitizers. When combined with other functional materials, they can regulate cellular reactive oxygen species, macrophage phenotypic transformation, bone resorption, osteogenesis, and mineralization, providing a new paradigm for bone tissue engineering. This study reviewed the progress in biomedical research progress on MOFs in diseases such as bone defects, osteoarthritis, osteoporosis, bone tumors, osteomyelitis, and periodontitis over the past five years.

The greatest advantage of materials based on MOF composites in the treatment of bone diseases is that they can be synthesized into different morphologies as needed, and they have shown improved drug solubility, loading, and release; targeting ability; ROS regulation ability; and pH and photothermal response, among other benefits. These factors help to significantly overcome the limitations of existing drugs and also increase the possibility of precise treatment. While the multifunctional properties of MOFs underscore their promising potential in bone-related biomedical applications, a comprehensive and critical evaluation across different MOF families reveals intricate variations in their performance, biocompatibility, and long-term suitability. ZIFs, for instance, have shown great chemical stability and good drug-loading capabilities, but their arbitrary breakdown routes can let imidazole derivatives out, hence generating questions about cytotoxic effects that might endanger cell survival [152]. While UiOs are known for their high thermal stability and chemical stability, emerging evidence suggests potential risks related to their long-term accumulation in tissues [153], which necessitates a deeper inquiry into their biodegradation kinetics and clearance mechanisms. Similarly, the MIL and HKUST frameworks have been shown to facilitate the effective delivery of therapeutic contents, but their degradation products may trigger tissue toxicity [154]. Therefore, it is necessary to conduct strict and systematic research under well-controlled experimental conditions to clarify the relationships between the physicochemical properties, biological interactions, and therapeutic effects of MOFs. This is crucial for determining the translational potential of MOFs in bone tissue engineering and regenerative medicine.

To date, there have been relatively few clinical experiments using MOFs as bone materials. Before MOF-based treatment methods can be translated into clinical bone regeneration applications, several practical challenges must still be addressed. The first issue is the safety of MOFs in vivo. At present, animal experiments are conducted through in situ implantation and blood injection. The organ distribution, metabolism, and release kinetics of MOFs and other functional materials in the body have not received much attention. These factors all determine the potential of MOFs in clinical applications. Therefore, it is necessary to establish a gold standard for biosafety to ensure the safety and efficacy of MOFs. The bottleneck of transformation lies in the unclear complex interaction relationship between the degradation kinetics of MOFs and the physiological microenvironment. In vitro models have shown that the controlled decomposition of biological systems, enzyme activity, and mechanical stress can produce unpredictable dissolution patterns, thereby altering the drug released dose and safety of drugs. The lack of correlation between pharmacokinetics and pharmacodynamics exacerbates this uncertainty. In addition, existing research lacks standardized protocols to track the biological distribution, metabolic pathways, and renal/liver clearance mechanisms of metal ions. Future research must focus on the frameworks of biocompatible metals (such as iron and calcium) and their combination with linkers that can be safely and predictably degraded in physiological environments. Combining complex surface modifications—such as bionic coatings that imitate natural tissue interfaces—can significantly prevent immune responses, thereby enhancing therapeutic effects and biocompatibility.

The second issue is the biological mechanism of MOF composites. Most studies have verified the roles of such composites both in vivo and in vitro, but they have not yet clearly explained the molecular switches and signaling pathways through which they exert their effects. These can be further explored through methods such as transcriptomics and proteomics.

The third issue is the fine regulation and standardization of MOF synthesis. The scalability of manufacturing comes at the cost of repeatability, where changes in particle size and morphology can alter performance. In clinical applications, stable and effective MOF complexes are required. Different laboratories may produce MOF NPs with different particle sizes and morphologies when using the same parameters, and all of these factors may affect the standardized production of MOFs. Therefore, it is necessary to develop more accurate MOF preparation methods and establish standards for the processing methods of MOF composite materials for clinical use.

In summary, the application of MOFs in bone diseases brings opportunities and challenges. By addressing the application limitations discussed above, MOFs are expected to become a key type of nanomaterial in the biomedical field, especially in advancing the treatment of bone-related diseases. In addition to their scientific prospects, the continuous development and clinical translation of MOF-based therapies may generate significant public health benefits through more effective, targeted, and personalized treatment strategies. These materials have the potential to shorten treatment time, reduce medical costs, and improve the quality of life of patients, especially among the aging population with an increasing prevalence of bone diseases. Furthermore, MOF research has promoted interdisciplinary collaboration between materials science, biology, and clinical medicine, enriching the scientific community and accelerating the development of next-generation bone defect treatment methodologies. With the progress in research, MOFs can also serve as a fundamental platform for the wider application of regenerative medicine and precision medicine, contribute long-term value to society, and shape new directions for biomedical innovation.

## Figures and Tables

**Table 1:** The main types of MOFs and their function for disease therapy.

Classification	Main Functionalization Methods	Main Strengths	Main Weaknesses	Impact on Osteogenesis	Impact on Antibacterial Activity	Impact on Biocompatibility
Drug carriers	In situ formation; post synthesis	Diverse design; high loading capacity; adjustable size	Unknown toxicity; low solubility; unknown homogeneity of structure	Promotes osteogenic differentiation; sustained release enhances bone repair [30,31]	Limited direct antibacterial effect unless combined with antibiotics [72,73]	High biocompatibility due to natural drug carriers; risk of inflammation if uncontrolled release [72,73]
Metal ion donors	In situ formation	Biologically active metal ions as coordination sites	Uncontrollable release kinetics and dose	Zn^2+^ upregulates RUNX2/ALP; Mg^2+^ enhances angiogenesis; supports mineralization [74,75]	Zn^2+^ disrupts bacterial membranes; Cu^2+^ induces ROS for pathogen elimination [76]	Cytotoxicity at high ion concentrations; requires controlled release kinetics [48]
Nanozymes	In situ formation; post synthesis	Low cost; high stability; excellent tolerance compared with nature enzyme	Unknown metabolic toxicity; long-term biocompatibility; immune responses	Scavenges ROS to reduce oxidative stress; promotes bone formation [61,62]	Mimics enzyme activity (e.g., catalase) to degrade bacterial biofilms [77]	May trigger immune responses if not surface-modified; Requires precise activity regulation [53]
Phototherapeutic agents	In situ formation; post synthesis	Small side effect on target tissue	Unknown biocompatibility; light transmission; targeted aggregation of MOFs, etc.	Photothermal stimulation upregulates osteogenic genes (e.g., COL1, OCN) [69,70]	ROS generation under light eliminates pathogens [78]	Depends on external stimuli (light/US), limiting deep-tissue applications; potential thermal damage if misapplied [79,80]

**Table 4:** Summary of the application of MOF-based composite materials for the treatment of osteoporosis.

MOF Composites	Form of the Composites	Primary Role of MOFs	Loaded Drugs and Loading Efficiency	Metal Ions/Clusters	Cellular Biological Functions	Mechanism	In Vivo Effects	Refs
[Sr(H_2_O)_3_(H_2_PXBP)]; [SrCa(H_2_O)_3_(H_2_PXBP)]	Scaffold	Released ions	/	Ca^2+^, Sr^2+^	Cell growth	/	/	[130]
Self-sacrificial MOFs	Nanoparticle	Drug carrier	Etidronate, pamidronate, alendronate, neridronate)	Mg^2+^, Ca^2+^	Cell growth	/	/	[131]
{[Ca(Cdcbp)]·2H_2_O}n MOF	Compound	Released ion	/	Ca^2+^	Promoting osteogenesis	/	Promoting mineralization	[132]
AHT-Ce/Sr MOF	Implant	Released ion	/	Ce^3+^, Sr^2+^	Reducting ROS; enhancing osteoblast differentiation; improving mitochondrial division and autophagy	Activating AMPK signaling	Promoting bone formation	[134]
ZIF8-NaHCO_3_@Cas9	Nanoparticle	Drug carrier	RANKL-CRISPR/Cas9 plasmids (~80%)	Zn^2+^	Reducting ROS; inhibiting bone resorption; promoting osteogenesis; delaying cell senescence	/	Promoting mineralization; delaying senescence	[135]

**Table 5:** Summary of the application of MOF-based composite materials for the treatment of periodontitis.

MOF Composites	Form of the Composites	Primary Role of MOFs	Loaded Drugs	Metal Ions/Clusters	Cellular Biological Functions	Mechanism	In Vivo Effects	Refs
Mino@ZIF-8	Nanoparticle	Drug carrier	Minocycline hydrochloride (~7.9%)	Zn^2+^	Relieving inflammation; protecting mitochondrial function	Enhancing AKT/GSK3β/NRF2 pathway	Reducing alveolar bone resorption; improving bone density	[75]
PP/PDA/ZIF-8	Membrane	Mechanical properties	/	Zn^2+^	Promoting osteogenesis	/	/	[138]
DZIF@PGel	Hydrogel	Drug carrier	Dexamethasone (~19.2%)	Zn^2+^	Antibacterial; relieving inflammation; inhibiting bone resorption; promoting osteogenesis	/	Promoting mineralization	[139]
L-MOF-agomir	Implant	Drug carrier	miR-27a (93–98%)	Zn^2+^	Antibacterial; promoting M1-to-M2 macrophage polarization; relieving inflammation; promoting osteogenesis	Metabolic shift from glycolysis to OXPHOS	Inducing macrophage to M2 polarization; relieving peri-implantitis bone resorption	[140]
SFD/CS/ZIF-8@QCT	Hydrogel	Drug carrier	Quercetin (~13.51%)	Zn^2+^	Antibacterial; relieving inflammation; promoting M1-to-M2 macrophage polarization; promoting osteogenesis	Activating PI3K-Akt signaling pathway	Repairing alveolar bone defects	[141]
Car@UiO-66; Thy@UiO-66	Nanoparticle	Drug carrier	Carvacrol (~79.60%); Thymol (~79.65%)	Zr^4+^	Antibacterial; relieving inflammation; promoting M1-to-M2 macrophage polarization; promoting vascularization; promoting osteogenesis	/	Facilitating bone defect healing	[72]
HMUiO-66-NH_2_	Nanoparticle	Nanozyme	/	Zr^4+^	Reducting ROS; inhibiting bone resorption; promoting osteogenesis	Promoting Wnt and TGF-β signaling pathways	Facilitating bone defect healing	[142]
CuTCPP-Fe_2_O_3_	Ointment	Photosensitizer	/	Fe^3+^, Cu^2+^	Antibacterial	/	Reducing alveolar bone loss; reducing tissue inflammation; promoting angiogenesis; alleviating periodontitis	[73]
CSBDX@MOF	Hydrogel	Drug carrier	Gallic acid	Mg^2+^	Antibacterial; Relieving inflammation; promoting M1-to-M2 macrophage polarization; inhibiting bone resorption; promoting osteogenesis	/	Reducing alveolar bone loss; enhancing collagen deposition	[143]
